# Characterising neovascularisation in fracture healing with laser Doppler and micro-CT scanning

**DOI:** 10.1007/s11517-013-1100-3

**Published:** 2013-07-24

**Authors:** W. Macdonald, S. J. Shefelbine

**Affiliations:** 1 Department of Bioengineering, Imperial College, South Kensington, London, SW7 2AZ UK; 2 Department of Mechanical and Industrial Engineering, Northeastern University, Boston, MA 02115 USA

**Keywords:** Fracture healing, X-ray micro-tomography, Angiogenesis, External fixators, Laser Doppler flowmetry

## Abstract

Vascularity of the soft tissues around a bone fracture is critical for successful healing, particularly when the vessels in the medullary canal are ruptured. The objective of this work was to use laser Doppler and micro-computer tomography (micro-CT) scanning to characterise neovascularisation of the soft tissues surrounding the fracture during healing. Thirty-two Sprague–Dawley rats underwent mid-shaft osteotomy of the left femur, stabilised with a custom-designed external fixator. Five animals were killed at each of 2, 4 days, 1, 2, 4 and 6 weeks post-operatively. Femoral blood perfusion in the fractured and intact contralateral limbs was measured using laser Doppler scanning pre- and post-operatively and throughout the healing period. At sacrifice, the common iliac artery was cannulated and infused with silicone contrast agent. Micro-CT scans of the femur and adjacent soft tissues revealed vessel characteristics and distribution in relation to the fracture zone. Blood perfusion dropped immediately after surgery and then recovered to greater than the pre-operative level by proliferation of small vessels around the fracture zone. Multi-modal imaging allowed both longitudinal functional and detailed structural analysis of the neovascularisation process.

## Introduction

Fracture healing of bone is a complex process affected by systemic, biological and mechanical factors, the combination of which can lead to successful and complete repair of a fracture, or deficiencies of which can cause delayed healing or even non-union.

Angiogenesis is an important early phase of fracture healing, leading to invasion of the initial haematoma by fine vessels and subsequent conversion into soft callus. Impaired angiogenesis can compromise fracture healing [8, 14], whereas enhanced vascularity is known to improve fracture healing [37, 43]. Neovascularisation of the haematoma occurs from the adjacent soft tissues [4, 9, 10, 13], especially when the medullary supply has been disrupted [12].

Neovascularisation in fracture healing has been analysed with a wide variety of techniques. Standard histology uses staining to visualise tissue types or tissue components (such as smooth muscle or endothelial cells) in the fracture callus and surrounding tissue [3, 21, 32, 42]. Immunohistochemistry techniques label proteins particular to blood vessels, such as VEGF and CD31, to identify regions of angiogenesis and quantify small vessels [22, 28, 36]. Capillary proliferation around the fracture site has been demonstrated by micro-angiography [29], and vascular budding adjacent to the fracture defect was revealed with contrast perfused histological sections [23]. Electron microscopy [21, 23, 38, 46] and intra-vital microscopy [47, 49] provide high-resolution images that can indicate fine details of the angiogenesis process, such as changes in cell morphology, gene expression or endothelial cell activity. These techniques can indicate fine details of the vessel structure and distribution, but most are two-dimensional and static, providing little quantitative information on blood flow.

Detailed three-dimensional structural information is provided by CT scanning with the vessels perfused with contrast resin at killing [2, 34], or in vivo [20, 26, 30, 31], but these are often limited snapshots at particular time points and provide no information of the time course of neovascularisation. For example, Tomlinson and associates [40] used silicone contrast perfusion of vessels and micro-computer tomography (micro-CT) scanning of forelimb stress fractures at 3 and 7 days after loading-induced stress fractures. However, scanning in vivo can give contrast and partial filling difficulties [7].

Measures of blood flow or blood perfusion are possible with probe-based laser Doppler [6, 15–17], ultrasound [35], radioactive tracer clearance [1, 33, 45, 48] or functional CT [18–20, 39]. These functional measures must be performed in vivo and provide temporal analysis of the neovascularisation process throughout the fracture healing period. Probe laser Doppler measures at discrete points (sometimes several across a region) and tracer clearance can be used to provide a regional integral of the measured flow. Laser Doppler scanning, however, scans continuously over a region and provides 2D surveys of blood perfusion, which can be used to identify vessels and regional flow patterns.

In this study, we combined laser Doppler scanning (for longitudinal percutaneous information) and micro-CT imaging (for end-point three-dimensional structural information) to investigate the location and extent of neovascularisation of the soft tissues around the fracture gap during healing.

## Methods

Thirty-two Sprague–Dawley rats (350–500 g; 12–20 weeks of age) underwent left mid-femoral osteotomy fixed with an external fixator. All procedures were approved by the Imperial College, London, ethical review process and were strictly conformed to the Animals (Scientific Procedures) Act 1986 UK Home Office guidelines. Experiments were performed under a Home Office Licence PPL 70/6472 (the conditions of which also fulfil the US NIH guide for the care and use of laboratory animals).

**Fig. 1:**
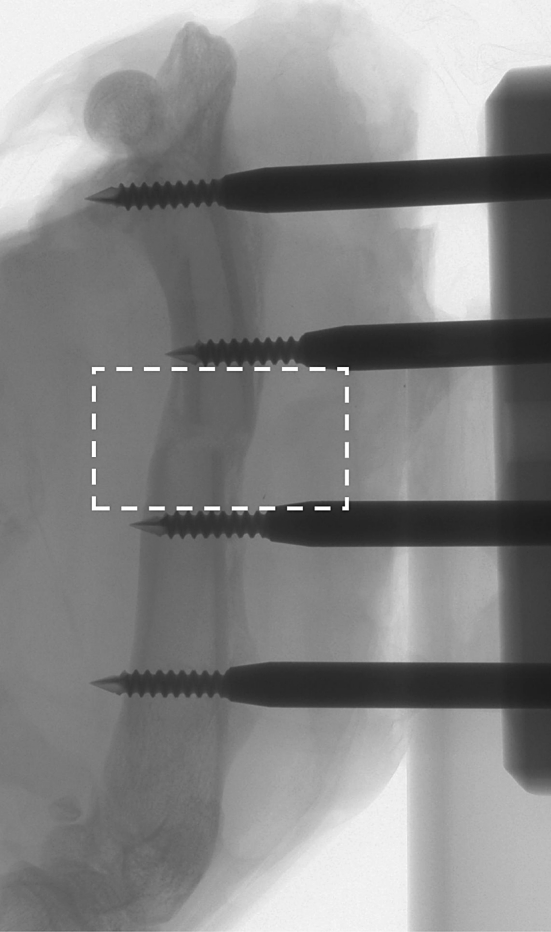
Radiograph of healed fracture (and fixator) sacrificed 6 weeks after fracture. The fracture zone is outlined

Inhalation anaesthesia was induced in an induction chamber (4 % isoflurane in oxygen at 2 L/min; IsoFlo, Abbott Laboratories Ltd., Maidenhead, SL6 4XE, UK), the animal was transferred to a rat mask and anaesthesia was maintained with 1 L/min oxygen and isoflurane varied as appropriate between 1.5 and 3 % to maintain full anaesthesia and analgesia but to allow optimal recovery. Prophylactic antibiotics (enrofloxacin; Baytril 0.05 %, Bayer plc. Newbury, RG14 1JA, UK) and analgesia (buprenorphine; Vetergesic 0.3 mg/ml.; Reckit Benckiser Healthcare (UK) Ltd, Hull, HU8 7DS, UK) were administered in appropriate doses; both thighs were shaved and laser Doppler scanned (model LDI2-HR, Moor Instruments plc, Axminster, England). The animal was then prepared recumbent on its side on a heating mat, surgically draped and skin disinfected for surgery. The left femur was exposed through a lateral skin incision and blunt dissection, four stainless steel fixation pins (1.25 mm thread diameter) were inserted transversely 7 mm apart along the femoral length and the femur was cut transversely mid-shaft using a fine hand saw (Fig. 1). The muscle layers and skin were closed (and sutured with resorbable suture material), the pin shafts were passed retrograde through the skin and a custom-designed unilateral fixator was assembled to the pin ends. After rehydration by injection of two 2 ml aliquots of sterile water subcutaneously, the animal was awoken with administration of pure oxygen through the anaesthesia equipment under observation and once fully awake was returned to standard housing. All animals were housed individually with analgesic (rimadil) and antibiotic cover (enrofloxacin 0.05 %) in the drinking water for 4 days. Thereafter, the animals were housed in threes and allowed water and standard laboratory feed *ad libitum*.

The full medial aspect of both thighs was laser Doppler scanned (2.5 cm × 2.5 cm) at a resolution of 0.1 mm, pre- and post-operatively and at 1, 2, 4 and 7 days and thence weekly until sacrifice. Animals were killed at 2 and 4 days post-operatively, and at 1, 2, 4 and 6 weeks post-injury (*n* = 5 per group).

At sacrifice, the anaesthetised animal was laser Doppler scanned, and then the common iliac artery was cannulated, heparinised and infused with silicone contrast agent (Microfil MV-120, Flow Tech Inc., Carver, Mass. USA). The animal was immediately euthanised with pentobarbital, and the hindquarters were harvested and fixed in 10 % neutral-buffered formalin solution for at least 2 weeks.

**Fig. 2:**
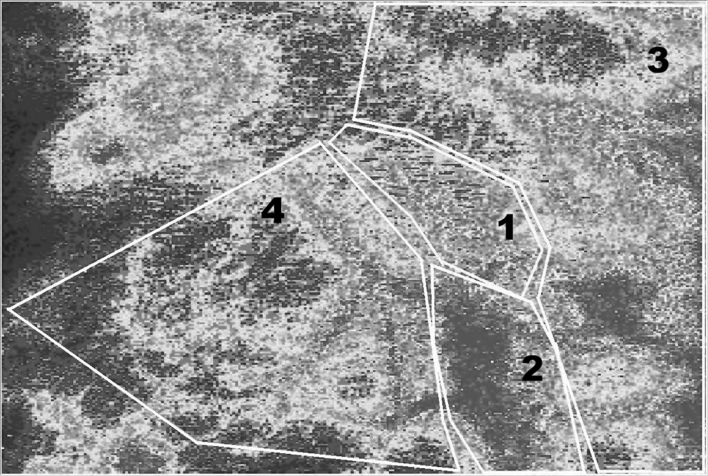
Laser Doppler scan with four regions of interest identified: *1* femoral artery, *2* distal femoral artery, *3* cranial region overlying the fracture zone and *4* caudal region

From laser Doppler scans, the femoral artery was identified by its bifurcation and location, and four regions of interest were analysed: (1) femoral artery, (2) distal femoral artery, (3) the zone cranial to the artery (adjacent to the fracture) and considered to be the tissue most probably involved in neovascularisation of the fracture site [13, 23, 29] and (4) the zone caudal to the artery (Fig. 2). Proprietary software (Moor LDI V5.1, Moor Instruments plc, Axminster, England) computed mean and maximum perfusion in each region; the mean values were used in subsequent analysis. Daily variations in perfusion due to depth of anaesthesia or body temperature were determined by calculating the change of perfusion in each region in the contralateral leg relative to the pre-operative value in that region (DailyVar). We divided the perfusion values of the fractured leg by this parameter to account for fluctuations not associated with the neovascularisation process. Mean perfusion measures (corrected for daily variations) were then expressed as a percentage change from the pre-operative value for the fractured leg. Inter-operator repeatability of the laser Doppler analysis was determined by three separate investigators analysing 20 separate scans independently. Intra-operator repeatability was determined by one-operator repeating analysis on 20 scans four times.

**Fig. 3:**
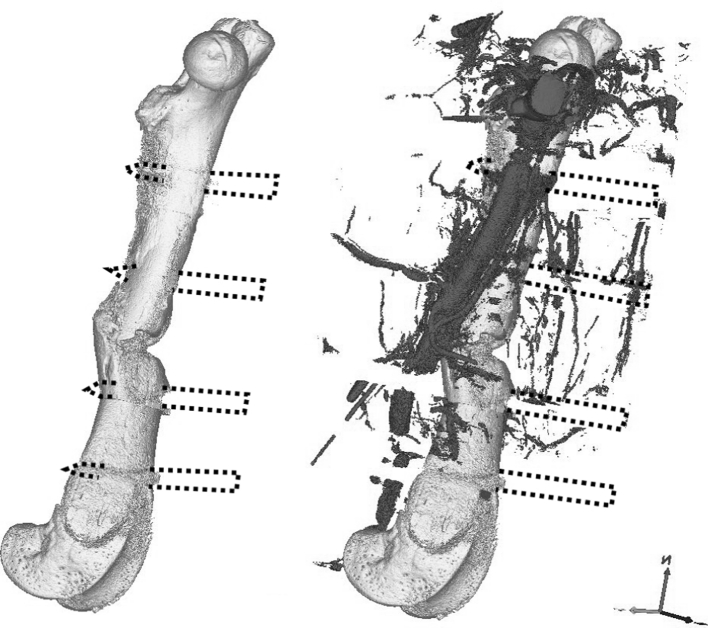
Three-dimensional reconstruction of μ-CT of bone 6 weeks after fracture (*medial view*). Pins were left in during scanning to preserve fracture morphology, resulting in artefacts at the level of the pins (gaps in the scan). This did not affect characterisation of vessels in the fracture gap. **a** Whole bone without vessels (the united fracture site can be seen mid-shaft, and the pin locations are indicated by *dotted lines*). **b** Whole bone and vessels (the femoral artery is clearly seen running across the medial aspect of the central zone of the femur)

Micro-CT scanning of the limb-fixator constructs and intact contralateral femora was performed at 180 kV and 133 mA and a resolution of 21 microns (HMX ST225, X-Tek Systems Ltd. Tring, UK). The excised femora were typically 30 mm in length and thus consisted of about 1,450 slices. The CT scans of both limbs were reconstructed to show the bone and the vessels (Fig. 3). The steel fixator pins caused some artefactual whiteout of the scans at their level, masking some of the vascular regions on those sections, but the fracture zone (which is the region of interest) was between the middle pins. Scans of fractured limbs were therefore analysed just between the pins on either side of the fracture site (proximally and distally, pins number 2 and 3), a length of about 8 mm. Intact limbs were analysed over the mid-third of the femur, which corresponded to the same level as the fracture region on the contralateral limb. The image stack of the scan was then thresholded automatically (“Trainable Segmentation”, Fiji Image Manipulation Software, http://fiji.sc/wiki/index.php/Fiji) to identify four regions: bone, vessel, tissue and air; the contrast medium-filled vessels displayed CT density midway between bone and soft tissue. The scans were binarised and manipulated to smooth and cohere the vessel regions and minimise all other regions. An automated particle counter (Fiji) was then used to count the vessels on each cross-section, the total cross-sectional (vessel) area and area fraction for each section, and the size and number of vessels in each slice were output to a spreadsheet. These could then be expressed as graphs of vessel number. The particle counter also reported the size of every particle counted (cross-sectional area of each vessel at each slice) in voxels, which was recorded, and the distribution of sizes was characterised in decades (1, 2, 5, 10, 50, 100, 500, 1,000 and 5,000 voxels, corresponding to vessels of cross-sectional area of less than 20, then approximately 30, 50, 70, 150, 200, 500 and 1,000 μm^2^). The proportion of small vessels was then calculated as the number of vessels whose cross-section was two voxels or less, summed for all slices in the fracture region divided by the total number of vessels in that region.

Statistical analysis of the perfusion data was performed using one-way analysis of variance (ANOVA) and also a multilevel (growth) model in SPSS (IBM SPSS Statistics, version 20^©^ 2011, IBM Corp. New York, USA). The growth model was used to analyse the raw perfusion measures from regional analysis considering the effects of time under a linear and quadratic model and using the corresponding measures in the intact leg (Intact), cranial (Cranial) and Cranial Intact and body temperature measures as covariates. Anova was used to test for differences between the fractured side and intact side measures. Statistical analysis of the repeatability studies was undertaken using Spearman’s correlation in SPSS.

Vessel density (vessel count) and vessel size in the study (fractured) limb were compared to those in the contralateral (intact) limb for the same region and at the same time point using paired Student’s *t* tests with the Bonferroni correction.

## Results

One cannulation failed, preventing contrast infusion, and two animals’ scans were compromised by wound difficulties (although they were still available for analysis). One longer term animal developed respiratory distress and was killed 1 week early (at 5 weeks). Therefore, 31 valid animals were micro-CT analysed at 2 days (*n* = 4), 4 days (*n* = 5), 6 days (1), 1 week (*n* = 5), 2 weeks (*n* = 6), 4 weeks (*n* = 5), 5 weeks (1) and 6 weeks (*n* = 4). All 6-week animals appeared successfully healed on radiographic projection (Fig. 1).

Inter-operator repeatability studies of the laser Doppler analysis gave a Spearman’s correlation coefficient of *r* = 0.88 for femoral artery regions and 0.83 for the cranial region. Spearman’s correlation coefficients for intra-operator repeatability were 0.96 and 0.94 for the femoral artery and cranial region, respectively.

**Fig. 4:**
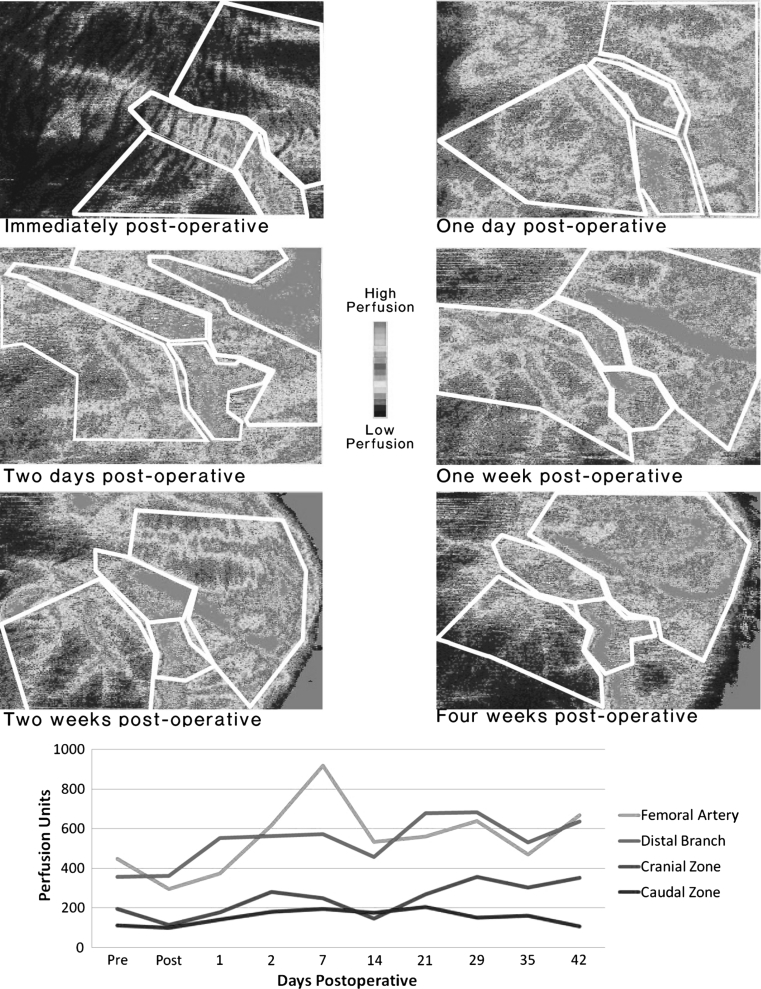
Laser Doppler scans at successive time points in a typical subject (*above*) and the corresponding plot of mean perfusion by zones

**Fig. 5:**
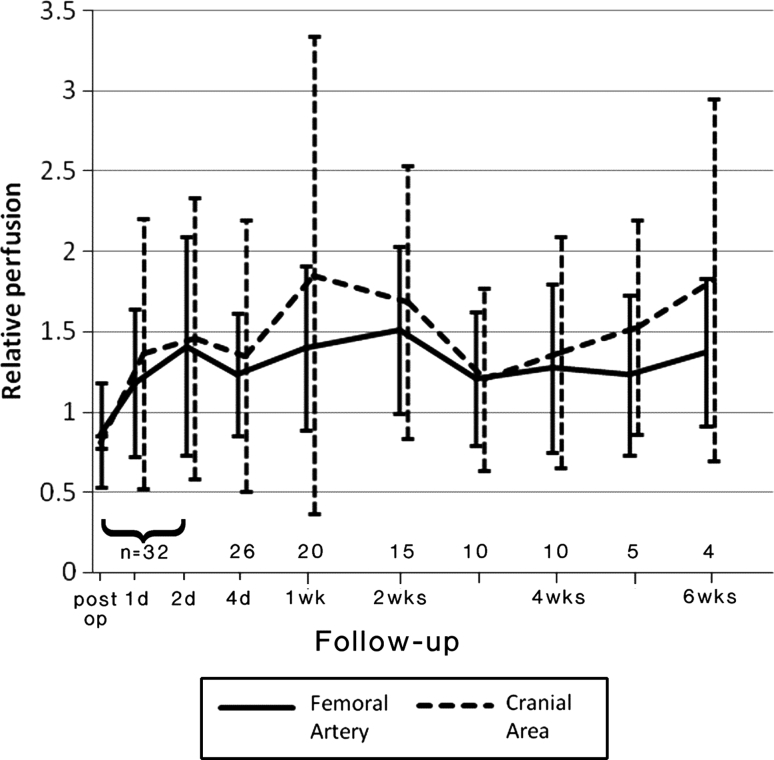
Perfusion (relative to pre-operative measures) in femoral artery region and cranial region (adjacent to fracture); Pooled data for all animals and all time points

Blood perfusion in the femoral artery (region 1) and the cranial region (region 3, which is adjacent to the fracture region) dropped immediately after operation, increased greatly from 4 days post-operatively to 2 weeks and then declined. The two other regions (2 and 4) showed similar trends to a lesser degree (Fig. 4). The mean regional perfusion values over all animals also demonstrated this trend (Fig. 5).

Statistical analysis confirmed these observations; multilevel statistical analysis showed that time after fracture significantly predicted perfusion in the femoral zone of the fractured leg, *F*(1,169.8) = 4.042, *p* = 0.046, and the corresponding measures on the intact limb also significantly predicted perfusion [*F*(1,181.7) = 8.83, *p* = 0.003]. The dependence on time was significantly better represented by the quadratic model than a linear growth model [*χ* ^2^(1) 9.858, *p* < 0.05], and inclusion of the intact measure was highly significant in improving the model [*χ* ^2^(5) 21.213, *p* < 0.001]. Body temperature did not significantly predict perfusion [*F*(1,187.92) = 0.167, *p* = 0.664] nor was there significant interaction between time and the perfusion measures [*F*(1,137.6) = 1.165, *p* = 0.282].

Anova demonstrated a highly significant difference between the femoral (fractured limb) perfusion measure and the intact side (*F* = 24.5, *p* < 0.001), with post hoc comparisons using the least-significant difference (LSD) technique indicating significant differences between pre-operative perfusion and post-fracture measures at days 2–14: immediate post-fracture perfusion was highly significantly different (*p* < 0.001) from all time points except 35 days (*p* = 0.018 from pre-fracture perfusion). Perfusion at day one was highly significantly different from post-fracture perfusion, but not from the pre-fracture measure (*p* = 0.109).

Micro-CT scans indicated no clear changes in spatial distribution of the vessels over time of healing, with an even distribution of vessel densities throughout the region surveyed.

**Fig. 6:**
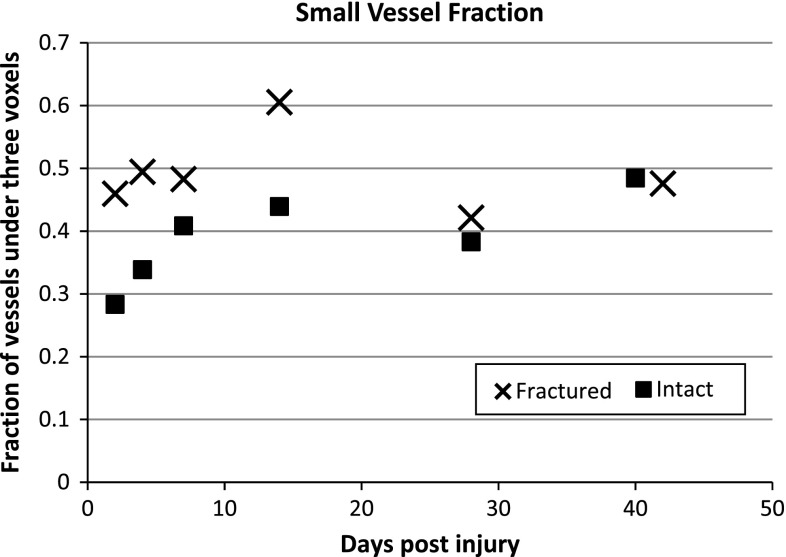
Small blood vessel fraction (number of vessels <3 voxels/total number of vessels) in the *fractured limb* and contralateral *intact limb*) (*p* = 0.021, Student’s paired *t* test)

Fractured limbs at all time points displayed highly significantly greater vessel densities (vessel number) than their contralateral intact limbs (*p* = 0.005, Student’s paired *t* test). The number of small vessels for each limb expressed as a fraction of the total number of vessels also showed a significantly greater proportion of smaller vessels on the fractured side (Fig. 6; *p* = 0.021, Student’s paired *t* test). This proportion also showed an increase from 2 days post-operatively to 14 days post-operatively, and then, the difference from the intact side diminished.

## Discussion

This study is the first to use two-dimensional laser Doppler scanning longitudinally throughout the healing period, to show an immediate post-operative decrease in perfusion adjacent to the fracture site, with a subsequent increase, especially throughout the first 2 weeks of healing. By combining this with micro-CT scanning, we have shown that this was achieved by an increase in the number and proportion of small vessels adjacent to the fracture zone. This elaborates the findings of Matsumoto et al. [25] who used laser Doppler scanning of the whole hind limbs of mice to identify perfusion changes (post-fracture and 2 weeks later) and histology to measure vessel density. Others have used single-point measures by laser Doppler probe to impute perfusion in whole regions, also reporting increased perfusion after fracture [32, 50].

Our laser Doppler measurements confirm that neovascularisation begins soon (2–14 days) after fracture, particularly in the soft tissues surrounding the fracture (cranial region of interest in laser Doppler scans). This indicates that neovascularisation of the tissue occurs in a spatially targeted region, not just through general limb perfusion, and is temporally organised to provide quick supply of nutrients and blood to the fracture site. The micro-CT vessel analyses indicate that this increased perfusion is achieved by more and finer vessels and not just by vasodilation of existing vessels. The vessel distributions around the fracture site (along the axis of the femur) were predominantly even and showed no consistent variation or trend towards one side of the fracture line or location. Although the pins were placed in the blunt dissected surgical approach, it is not possible to exclude any vascular response to this or the cortical fixation of the pins.

Similar time trends were reported by Melnyk et al. [27], who also found increased perfusion shortly after fracture followed by a decline, in their study of perfusion in fracture healing with soft tissue damage, using a probe-based transcutaneous laser Doppler flowmeter measuring single points at the fracture site and one centimetre distally and proximally. Perfusion near the fracture site was only clearly greater than preoperatively at 3 and 7 days, and 14 days slightly greater. Perfusion at the fracture gap lagged behind—greater perfusion occurred at 7 and 14 days after fracture.

Previous studies have shown similar trends in neovascularisation of soft tissue around the fracture callus, dating back to Gothman’s studies in the 1960s [9–12]. These studies established clinical practice for treatment of displaced fractures by underlining the benefit of soft tissue neovascularisation. However, neovascularisation analyses were limited to ex vivo assessment of 2D angiographs or histology, or single-point measures of perfusion. By combining laser Doppler scanning and micro-CT analysis, we were able to provide 3D structural and 2D functional measurements of vascularisation, showing that neovascularisation occurred by angiogenesis (new vessel formation) and not only vasculogenesis (vasodilation of existing vessels). Micro-CT scanning undecalcified bones enabled localisation of the vascularity with respect to the fracture gap, although segmentation and scan analysis were thereby made more difficult. Our images indicate that angiogenesis (as evident by number of small vessels) was evenly distributed along the fractured bone length.

Our micro-CT results correlate with the findings of Tomlinson et al. [41], who also used Microfil infiltration and micro-CT to identify vessels in osteogenic response to overloading. Using anti-angiogenic treatment, they showed that angiogenesis was significant in the increased vascularity at 3 and 7 days after loading-induced stress fracture-related osteogenesis. The use of Microfil to image vessels down to 10 microns was reported by Marxen et al. [24] and demonstrated by Vasquez et al. [44], working with similar 20 micron micro-CT resolution.

Neovascularisation of the soft tissues is critical as it provides a blood source for the healing fracture callus. In our current study, we were not able to visualise or quantify vessels directly within the fracture callus. The resolution and depth of penetration of the laser Doppler scan were insufficient to quantify the angiogenic processes in the callus. Though our micro-CT images most probably captured the intra-cortical vessels, the contrast between bone and vessels was insufficient to allow thresholding differentiation and segmentation. Further work will use histology and decalcified bones to examine the vessel structure in the bone and fracture callus.

Though variations in perfusion were large between animals (large standard deviations in Fig. 5), the temporal and spatial patterns of revascularisation were similar for all animals (statistical analysis, *p* < 0.05). Large variation in blood perfusion measurements is not uncommon due to the numerous internal and external factors that influence perfusion [5]. Sample size at each time point was small (*n* = 5) but comparable to similar studies [50], and larger sample sizes would probably not change the overall trends seen.

The depth of penetration for laser Doppler scanning depends on the tissue type. Our preliminary data indicated that laser Doppler scanning can detect flow through 5 mm of muscle tissue, and Melnyk et al. [27] report laser penetration of bone to 2 mm depth and skin/muscle to 6 mm. Perfusion images are weighted projections of perfusion as a function of depth (i.e., deeper vessels appear to have less flow). A few animals suffered skin nicks during shaving, which resulted in extremely high perfusion values despite relatively small skin injury. The injury caused by surgery was all on the lateral side of the leg, and scanning was performed on the medial side, minimising the skin effects of the surgical site.

Reed has shown that revascularisation at early time points is critical for ensuring healing [36]. In that rat model, delayed revascularisation resulted in atrophic non-union. Now that we have developed methods for functional and structural characterisation of neovascularisation, future work will examine methods for increasing vascularity of the soft tissue in these early time points.

Laser Doppler scanning is a non-invasive in vivo measurement causing minimal distress to the animal which can be used for longitudinal studies of treatment effects and responses. Micro-CT complements the functional measures provided with laser Doppler with 3D structural information of the vessel network. Further investigation using these modalities in fracture healing studies may enable correlation with other factors that promote the healing process. 
